# *Microsporum gypseum* Isolated from *Ailuropoda melanoleuca* Provokes Inflammation and Triggers Th17 Adaptive Immunity Response

**DOI:** 10.3390/ijms231912037

**Published:** 2022-10-10

**Authors:** Xiaoping Ma, Zhen Liu, Yan Yu, Yaozhang Jiang, Chengdong Wang, Zhicai Zuo, Shanshan Ling, Ming He, Sanjie Cao, Yiping Wen, Qin Zhao, Rui Wu, Xiaobo Huang, Zhijun Zhong, Guangneng Peng, Yu Gu

**Affiliations:** 1 Key Laboratory of Animal Disease and Human Health of Sichuan Province, College of Veterinary Medicine, Sichuan Agricultural University, Chengdu 611130, China; 2 China Conservation and Research Center for the Giant Panda, Chengdu 611800, China; 3 College of Life Sciences, Sichuan Agricultural University, Chengdu 611130, China

**Keywords:** *Microsporum gypseum*, lung, immune response, receptor, cytokine

## Abstract

*Microsporum gypseum* causes dermatomycoses in giant pandas (*Ailuropoda melanoleuca*). This study aimed to investigate the immune response of *M. gypseum* following deep infection. The degree of damage to the heart, liver, spleen, lungs, and kidneys was evaluated using tissue fungal load, organ index, and histopathological methods. Quantitative reverse transcription-polymerase chain reaction (qRT-PCR) detected the mRNA expression of receptors and cytokines in the lung, and immunofluorescence staining and flow cytometry, were used to assess immune cells in the lung. The results indicated that conidia mainly colonized the lungs and caused serious injury with *M. gypseum* infection. Furthermore, dectin-1, TLR-2, and TLR-4 played a role in recognizing *M. gypseum* cells. Numerous inflammatory cells, mainly macrophages, dendritic cells, polymorphonuclear neutrophils, and inflammatory cytokines (TGF-β, TNF-α, IL-1β, IL-6, IL-10, IL-12, and IL-23), were activated in the early stages of infection. With the high expression of IL-22, IL-17A, and IL-17F, the Th17 pathway exerted an adaptive immune response to *M. gypseum* infection. These results can potentially aid in the diagnosis and treatment of diseases caused by *M. gypseum* in giant pandas.

## 1. Introduction

*Microsporum gypseum*, a ubiquitous geophilic dermatophyte found worldwide, can infect humans [1] and many animals [2,3,4,5,6,7], including dogs, cats, horses, giant pandas, pigs, dromedary camels, gray wolves, and Bradypus variegates., mainly causing skin diseases and deep infections in immune deficient individuals. In 2013, a dermatomycosis case in a giant panda caused by *M. gypseum* was reported in Sichuan, China, with symptoms of dehairing, excoriation, vesicle, skin chapping, and nidus abscess [8]. When immunity is weakened, deep infection by dermatophytes leads to infiltration of the skin and subcutaneous tissue, and causes lesions of the lymph nodes, internal organs, and central nervous system [9]. Walter et al. [10] reported a case of granulation tissue in the ankle joint following an *M. gypseum* deep infection in a renal transplant recipient. However, knowledge gaps surrounding the immune system response to *M. gypseum* pose barriers to the comprehensive understanding of this infection.

The recognition and elimination of fungal pathogens mainly depends on immune phagocytes, especially macrophages, neutrophils, and dendritic cells (DCs), which engulf fungal cells and initiate antigen presentation. When the body is invaded by fungi, innate immunity is rapidly activated, and pattern-recognition receptors (PRRs) can recognize the pathogen-associated molecular patterns (PAMPs) of pathogens and activate a series of signaling pathways to trigger a natural immune response [11]. Current research indicates that PRRs mainly include C-type lectin-like receptors (CLRs), Toll-like receptors (TLRs), retinoic acid-inducible gene I (RIG-I)-like receptors (RLRs), and nucleotide-binding oligomerization domain (NOD)-like receptors (NLRs). CLRs and TLRs are the main PRRs on the surface of the immune and non-immune cell membranes. Dectin-1, TLR-2, and TLR-4 often serve as markers of PRRs activation. Dectin-1 assists the recognition of β-glucans on the surface of fungal cells to recognize fungi such as *Candida albicans* [12,13]. Ligand binding to Dectin-1 induces a series of cellular reactions, including the recognition and uptake of the ligand, the promotion of differentiation and maturation of immune cells, the activation of T-helper 1 (Th1) and Th17 pathways, and the release of arachidonic acid metabolites and a variety of cytokines and chemokines, including, IL-2, IL-10, CXCL2, TNF, IL-1β, IL-6, and IL-23 [13,14]. TLRs are associated with Th17 immune response. TLR-2 and TLR-4 induce the body to produce an immune response via recognition and binding to phospholipid mannan and α-mannan of the cell wall, respectively [15]. The deletion of TLR-2 and TLR-4 genes in mice reduces the recruitment of neutrophils and release of chemokines, thereby, reducing the ability to clear fungi [16].

Polymorphonuclear neutrophils (PMNs) [17], DCs [18], and macrophages [19] can produce pro-inflammatory cytokines to induce adaptive immune responses after being recognized by PRRs in response to dermatophyte infection. Dectin-1 and Toll-like receptors synergistically induce the production of IL-6, IL-23, IL-10, IL-1β, and TNF-α, thereby stimulating Th17 immune responses [20,21]. Mao et al. (2018) found that *Microspora canis* induced rapid production of IL-1β and active cathepsin B in both human and mouse cells, thereby, promoting antifungal responses *in vivo* [22]. The secretions from *M. canis* were vaccinated into peripheral blood mononuclear cells and induced a strong antibody response and an increase in interferon-γ (IFN-γ) mRNA levels [23]. *M. canis* induced the overexpression of PMN surface receptors, TLR-2 and TLR-4, induced the secretion of cytokines by PMNs [17,24], and promoted skin Langerin-expressing cells, contributing to the antifungal Th17 response *in vivo* [25].

Cellular immunity (CMI) of animals following a fungal infection plays a vital role in clinical recovery, and CD4+ T lymphocytes are essential. The CD4+ cell subset includes Th1, Th2, Th17, regulatory T (Treg) cells, follicular helper T (Tfh) cells, and Th9 [26]. Th1 cells mainly produce IFN-γ, resist infections by intracellular pathogens, and participate in autoimmune diseases. Th2 cells, in turn, mainly produce IL-4 and IL-13, which are involved in the allergic immune response. Th17 cells mainly produce IL-17A, IL-17f, and IL-22, which are present at mucosal sites and are prevalent in the immune response against extracellular bacteria and molds. The Th1 and Th17 pathways [11,27] are crucial immune responses against dermatophytes, such as infections by *Trichophyton rubrum* [28], *Arthroderma benhamiae* [29], *oral candidiasis* [30], and *Aspergillus fumigatus* [31]. 

To date, there are only a few reports on the immune response to deep infection by *M. gypseum*. The purpose of this study was to investigate the main target sites, pathological symptoms, and adaptive immune responses of *M. gypseum* in deep infections of the body. Here, the main target sites were determined by studying the organ index, tissue fungal burden, and histology following deep infection with *M. gypseum*. Additionally, immune cells recruited to colonization sites were identified, and the percentage of Th cells and cytokine gene expression induced by infected mice were assessed, providing a scientific basis for the effective prevention, diagnosis, and treatment of deep infections caused by *M. gypseum*.

## 2. Results

### 2.1. Murine Weight and Mortality

**Figure 1:**
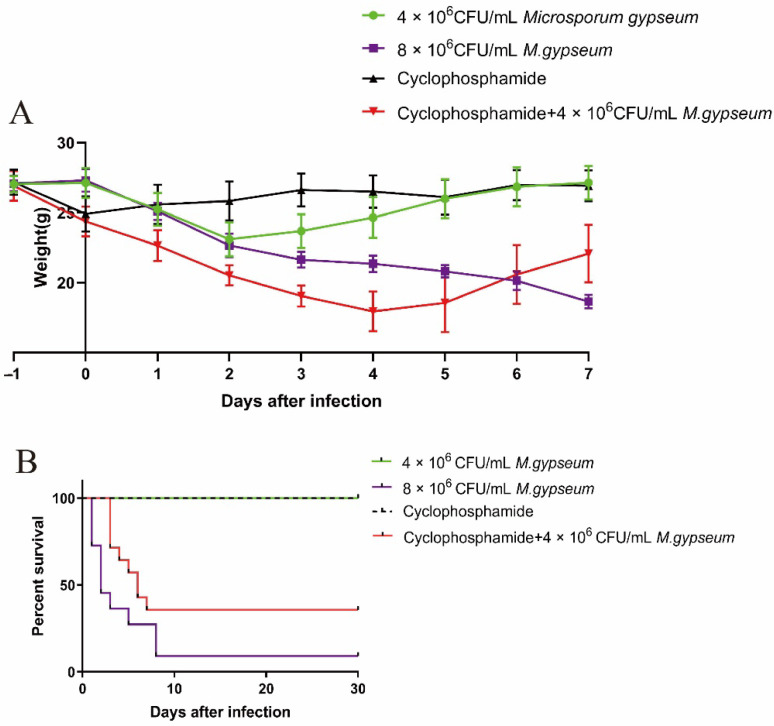
Weight and mortality of mice: (**A**) Weight change of mice after i.v. infection with *Microsporum gypseum*, cyclophosphamide was injected i.p. into two groups of mice on the day before infection with *M. gypseum* (15 mice per group); (**B**) Survival curves of mice after i.v. infection with *M. gypseum*, cyclophosphamide was injected i.p. into two groups of mice in the day before infection with *M. gypseum* (15 mice per group). The results are presented as mean ± SD and represent three individual experiments.

All the animals infected with *M. gypseum* exhibited dyspnea and weight loss. According to the results of two non-immunosuppressed mice injected with different concentrations of fungal suspension, *M. gypseum* induced weight loss, and higher concentrations were associated with greater weight loss as well as increased mortality. The high-concentration group required a longer time to recover weight. Infection of healthy and immunosuppressed mice with the same concentration of fungal suspension, indicated that immunosuppressed mice were more susceptible to *M. gypseum*, resulting in a more significant weight loss and slower recovery, and most importantly, a significantly increased mortality (Figure 1).

### 2.2. Tissue Fungal Burden

**Figure 2:**
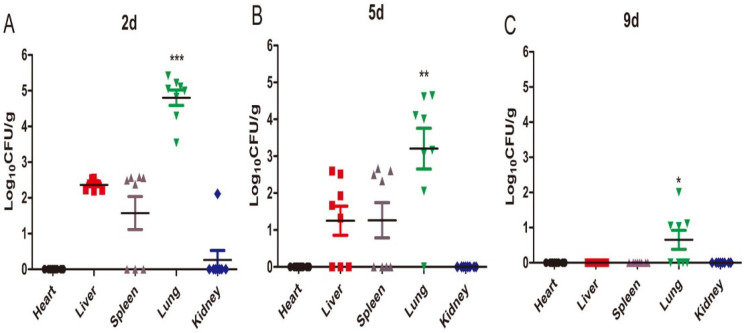
Tissue fungal burden (**A**–**C**). Means of fungal burdens in the heart, liver, spleen, lungs, and kidneys of mice (n = 8) on day 2, 5, and 9 after i.v. infection with *M. gypseum* (4 × 10^6^ CFU/mL). The results are presented as mean ± SD and represent three individual experiments (n = 8 mice per group). * *p* < 0.05; ** *p* < 0.01; *** *p* < 0.001.

The tissue fungal burden analysis results are shown in Figure 2. Overall, no fungi cells were found in the heart or kidneys at any given time. With increased inoculation time, the fungal burden in the liver, spleen, and lungs showed a decreasing trend. The highest fungal load was detected in the lungs, followed by the liver and spleen, at 2 days post infection (dpi) (*p* < 0.001) and 5 dpi (*p* < 0.01). Fungi were only detected in the lungs at 9 dpi. These results suggest that the fungi mainly colonized the lungs after *M. gypseum* infection.

### 2.3. Tissue Weight and Organ Index

**Figure 3:**
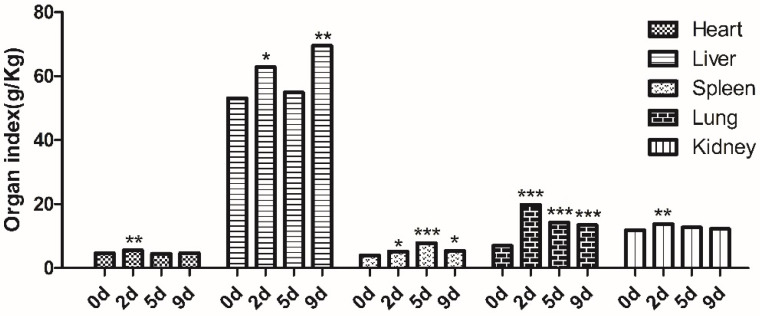
The organ index of heart, liver, spleen, lung, and kidney from mice (n = 8) on days 2, 5, and 9 after infection; i.v. infected with *M. gypseum* (4 × 10^6^ CFU/mL). The results are presented as mean ± SD and represent three individual experiments (n = 8 mice per group). * *p* < 0.05; ** *p* < 0.01; *** *p* < 0.001.

The heart, liver, spleen, lung, and kidney weights were determined at 2, 5, and 9 dpi and compared with those on day 0 (Figure 3). The organ index (OI) of the heart and kidney peaked at 2 dpi, and their OI values were significantly higher than those on day 0 (*p* < 0.01); however, at 5 and 9 dpi, significance was lost. The OI value of the liver peaked at 9 dpi, and at 2 and 9 dpi was significantly higher than that on day 0 (*p* < 0.05), but at 5 dpi it was not significantly higher than on day 0 with the value declining, presumably because the body’s immune system and invasive fungi were still in a state of confrontation and did not reach a balance. *M. gypseum* had a greater effect on the spleen and lungs than on the other three organs. The OI value of the spleen peaked at 5 dpi (*p* < 0.01), and at 2 and 9 dpi, it was significantly higher than that on day 0 (*p* < 0.05). After infection, the OI of the lungs from 2 dpi to 9 dpi was significantly higher than that on day 0 (*p* < 0.001). These results suggest that the lung was severely affected by *M. gypseum*, and the OI value increased, suggesting that there might be pathological changes, such as congestion, edema, or hypertrophy.

### 2.4. Histology 

**Figure 4:**
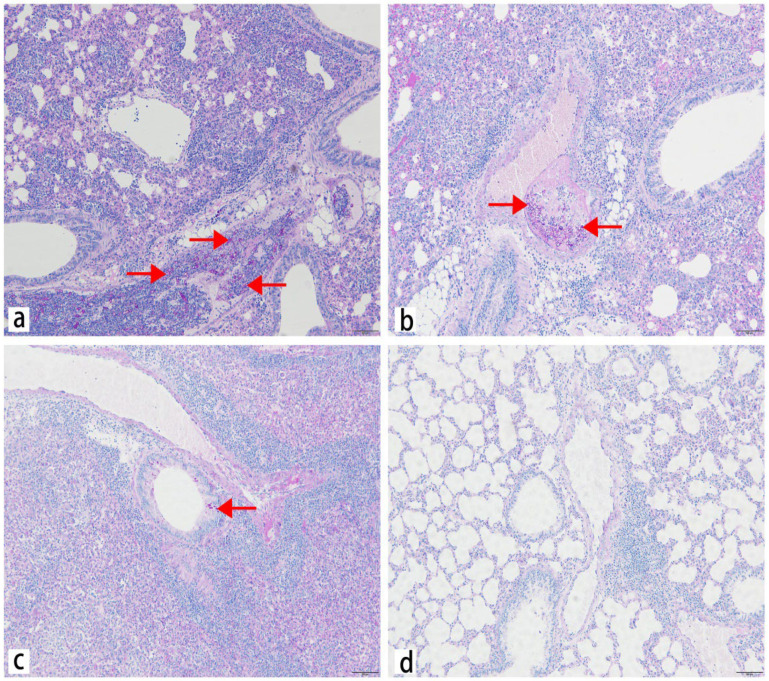
Representative PAS-stained sections of lung from mice on days 2 (**a**), 5 (**b**), 9 (**c**), and 14 (**d**) after infection; i.v. infected with *M. gypseum* (4 × 10^6^ CFU/mL). The fungal colonization in mice infected with *M. gypseum* after infection on days 2 (**a**), 5 (**b**), 9 (**c**) is shown. The alveolar septa and bronchus were colonized by dermatophyte arthroconidia in the lung. The spores are indicated with arrows. Scale bars, 500 μm.

To assess the presence and localization of *M. gypseum* to the lungs, spleen, liver, heart, and kidneys of the infected mice, tissues were stained with periodic acid-Schiff (PAS). The results showed that many conidia were detectable in the veins of lung tissue, and some conidia were detected in the alveoli septum at 2 dpi. Some conidia entered the bronchus at 5 dpi and budded at 9 dpi, but no hyphae were found in the lungs (Figure 4). Only a few conidia were present in the spleen, whereas no conidia were found in the liver, kidneys, and heart (Supplementary Figure S1A–D). These results indicated that a large amount of *M. gypseum* mainly colonized the lungs, which was consistent with the results of the tissue fungal burden experiment. 

**Figure 5:**
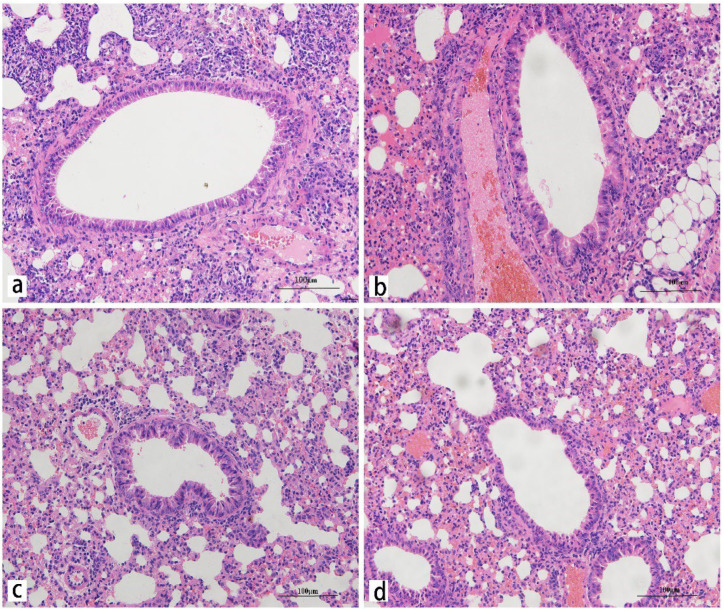
Representative HE-stained sections of lung from mice on days 2 (**a**), 5 (**b**), 9 (**c**), and 14 (**d**) after infection; i.v. infected with *M. gypseum* (4 × 10^6^ CFU/mL). Lung injury was mainly featured with destroyed alveoli, decreased number of alveoli, thickened alveoli septum and significant inflammatory cell infiltration; and exudation of fibrin was found in the alveolar on days 2 (**a**) and 5 (**b**) after infection. Scale bars, 100 μm.

To assess histological lesions in the heart, liver, spleen, lung, and kidney of the infected mice, tissues were stained with hematoxylin and eosin (HE). The lung was as solid as the liver. Lung injury mainly features destroyed alveoli, decreased alveolar numbers, thickened alveoli septum, and visible fibrin exudates in the alveoli. Different degrees of congestion and hemorrhage occurred in the lungs from 2 dpi to 14 dpi, with the most severe effects at 5 dpi. Extensive inflammatory cell infiltration was observed around blood vessels and lung tissue (Figure 5). Pathological damage in the lungs was more serious at 5 dpi, and some pathological damage was observed in the spleen and liver. Spleen injury featured trabecular hyperplasia, increased multinuclear macrophages, unclear boundary between the white and red pulp, and red pulp inflammatory cell infiltration (Supplementary Figure S2A). Liver injury included central venous congestion, hepatic cord structure disorder, and vacuoles of varying sizes in the hepatocyte cytoplasm (Supplementary Figure S2B). Kidney injury exhibited renal epithelial cell necrosis, renal interstitial congestion, and narrowing of the renal capsule (Supplementary Figure S2C). As the infection time increased, the lungs, liver, and spleen gradually recovered from the pathological sections. These results indicated that *M. gypseum* inflicted higher damage to lungs compared to other tissues. Congestion and hemorrhage occurred in the lung tissue, and the lungs were as solid as the liver, resulting in lung enlargement and weight gain, which was consistent with the results of the OI experiment.

According to the results of tissue fungal burden, OI, and histology, the lung was the main organ of *M. gypseum* attack; therefore, we subsequently studied the immune response of the lung following *M. gypseum* infection. 

### 2.5. Quantification of Receptor mRNA Levels in the Lung after M. gypseum Infection

**Figure 6:**
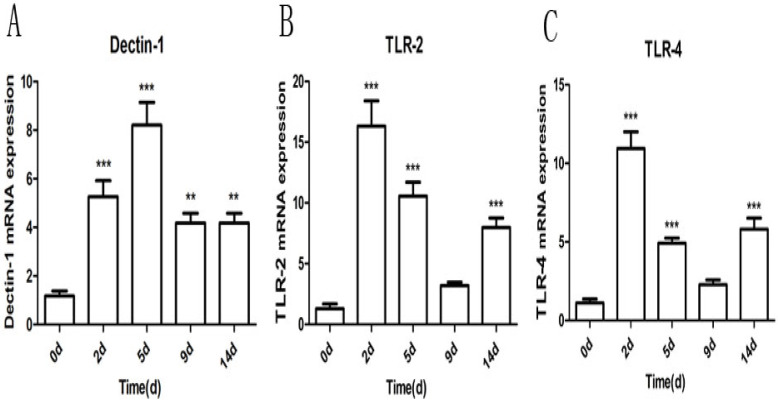
(**A**–**C**) *M. gypseum* (4 × 10^6^ CFU/mL)-induced expression of selected receptor genes (Dectin-1, TLR-2, TLR-4) detected by quantitative reverse transcriptase-polymerase chain reaction (qRT-PCR) in the lungs at 2, 5, 9, and 14 days after infection. The results are presented as mean ± SD and represent three individual experiments (n = 8 mice per group). ** *p* < 0.01; *** *p* < 0.001.

Receptor mRNA expression levels were determined at 2, 5, 9, and 14 dpi and compared with those on day 0 (Figure 6). Infection with *M. gypseum* induced a statistically significant upregulation of Dectin-1, TLR-2, and TLR-4 at 2, 5, and 14 dpi in the lung (*p* < 0.01); the peak of Dectin-1 emerged at 5 dpi, and TLR-2 and TLR-4 emerged at 2 dpi. At 9 dpi, the Dectin-1 expression levels were significantly higher than on day 0 (*p* < 0.01). There was no statistical difference between TLR-2 and TLR-4 on day 0, presumably because the immune system and invasive fungi were still in a state of confrontation and did not yet reach a balance. These results suggest that Dectin-1, TLR-2, and TLR-4 receptors play a role in recognizing invading *M. gypseum*.

### 2.6. Quantification of Cytokine mRNA Levels in the Lung after M. gypseum Infection

**Figure 7:**
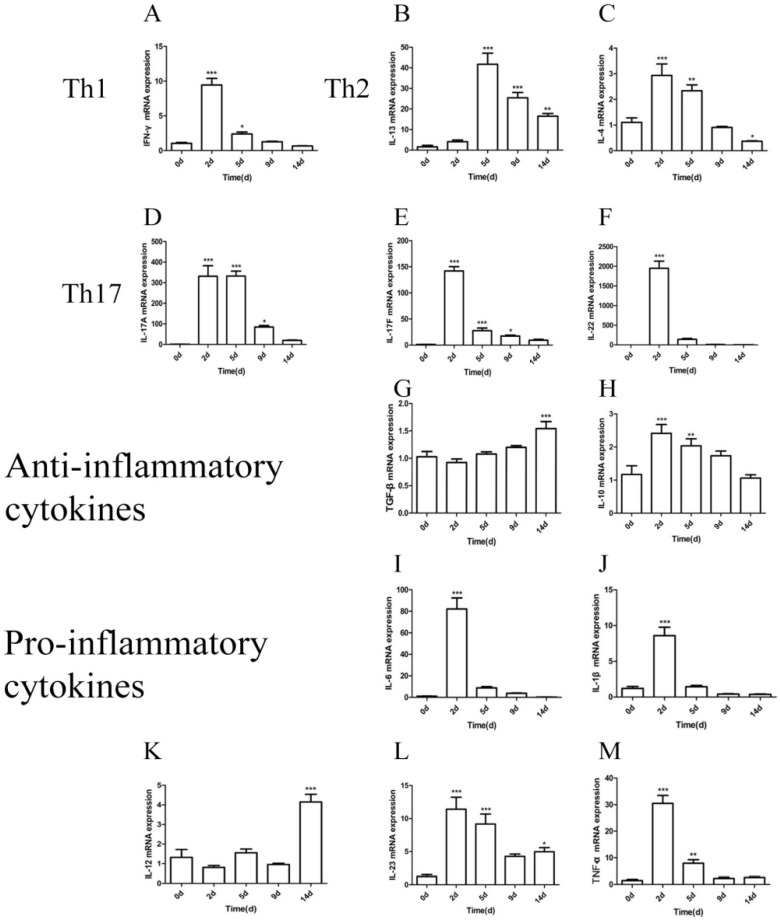
(**A**–**M**) *M. gypseum* (4 × 10^6^ CFU/mL)-induced expression of cytokines genes (Th1, Th2, Th17, anti-inflammatory cytokines, and pro-inflammatory cytokines) detected by qRT-PCR in the lung after infection on days 2, 5, 9, and 14. Th1: IFN-γ (**A**); Th2: IL-4 andIL-13 (**B**,**C**); Th17: IL-17A, IL-17F, and IL-22 (**D**–**F**); anti-inflammatory cytokines: TGF-β and IL-10 (**G**,**H**); pro-inflammatory cytokines: IL-6, IL-1β, IL-12, IL-23, and TNF-α (**I**–**M**).The results are presented as mean ± SD and represent three individual experiments (n = 8 mice per group). * *p* < 0.05; ** *p* < 0.01; *** *p* < 0.001.

Cytokine mRNA expression levels were determined at 2, 5, 9, and 14 dpi compared with day 0, and were all upregulated post-infection with *M. gypseum* (Figure 7). IFN-γ was significantly upregulated at 2 and 5 dpi, peaking at 2 dpi (*p* < 0.001). However, the instructive cytokine IL-12 was not significantly different from 2 to 9 dpi but was upregulated at 14 dpi (*p* < 0.001). This suggests that the Th1-associated cytokine was weakly expressed during the immune response and that a large amount of IFN-γ was not produced by the Th1 cells.

IL-13 was significantly upregulated at 5 dpi (*p* < 0.001), with a gradual downward trend from 5 to 14 dpi. IL-4 was significantly upregulated at 2 dpi (*p* < 0.001), gradually decreasing from 2 to 14 dpi, and was significantly downregulated at 14 dpi (*p* < 0.05). These results indicate that Th2-associated cytokine expression was gradually suppressed during the immune response.

Inflammatory-related cytokines are potent members of the Toll-like receptor signaling pathway and promote Th17 differentiation. The relative expression levels of IL-17A, IL-17F, and IL-22 were the highest (>100), followed by IL6 (>50) among all cytokines. The mRNA levels of IL-1β, IL-6, Il-10, IL-23, and TNF-α were significantly upregulated at 2 and 5 dpi, and peaked at 2 dpi (*p* < 0.001). IL-17A, IL-17F, and IL-22 were produced by Th17 cells, and their mRNA expression was significantly upregulated from 2 to 9 dpi, peaking at 2 dpi (*p* < 0.001). These results suggest that Th17-associated cytokines are highly expressed during the immune response.

IL-10 was significantly upregulated at 2 and 5 dpi, peaking at 2 dpi (*p* < 0.001). TGF-β was gradually upregulated from 2 to 14 dpi, peaking at 14 dpi (*p* < 0.001). The results indicate that Treg-associated cytokines were involved in the immune response.

### 2.7. Immune Cells Recruitment following M. gypseum Infection

**Figure 8:**
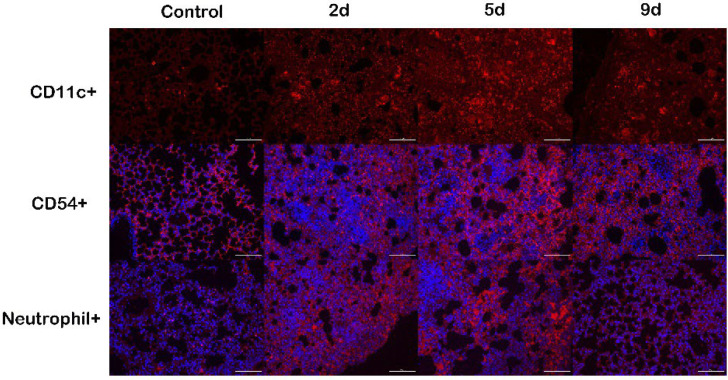
Immune cells were recruited in the lungs of mice infected with *M. gypseum* (4 × 10^6^ CFU/mL) on days 2, 5, and 9 after infection. Immunofluorescence staining revealed that polymorphonuclear neutrophils (PMNs), dendritic cells (CD11c, CD11c+), and macrophages (CD54, CD54+) were recruited mainly around the alveolar septum (red fluorescence increased). Scale bars, 100 μm.

Lung tissue immunofluorescence staining was performed to detect immune cell recruitment on 0, 2, 5, and 9 dpi (Figure 8). Immune cells were recruited to the lungs at 2, 5, and 9 dpi compared with day 0. Immunofluorescence staining revealed that large numbers of PMNs (neutrophils +), DCs (CD11c+), and macrophages (CD54+) were recruited to the alveoli septa and bronchi of *M. gypseum* infected mice. Accordingly, these immune cells were scarce in the lungs of control mice. These results suggest that forceful pulmonary innate immune responses were induced during *M. gypseum* infection. 

### 2.8. Th Cells Detection after M. gypseum Infection

**Figure 9:**
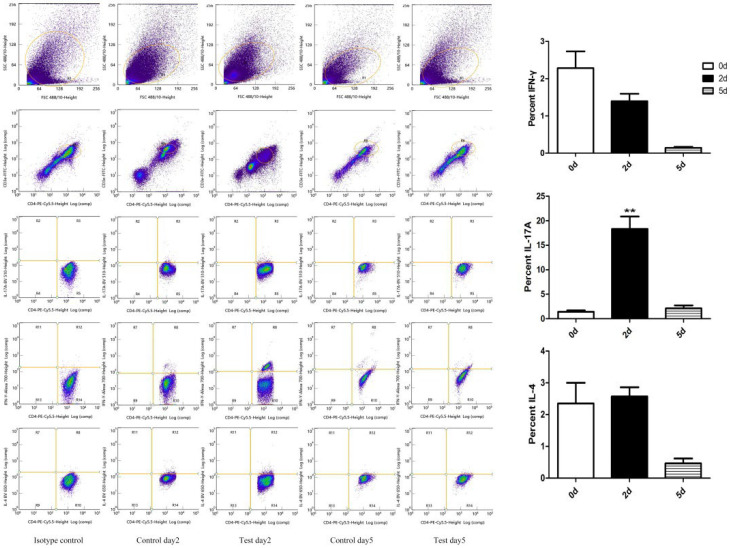
Th cells were recruited in the lungs of mice (n = 6) infected with *M. gypseum* (4 × 10^6^ CFU/mL) on days 2 and 5 after infection. We detected Th cells (Th1, Th2, and Th17) with the antibodies IFN-γ, IL-4, and IL-17A, respectively. The results are presented as mean ± SD and represent three individual experiments (n = 6 mice per group). ** *p* < 0.01.

Flow cytometry results indicated that Th cells were recruited to the lungs at 2 and 5 dpi compared with day 0. Th17 cells significantly increased at 2 dpi (*p* < 0.01), with no significant difference at 5 dpi. Th1 and Th2 cells did not differ significantly between 2 and 5 dpi (Figure 9). These results indicate that the Th17 signaling pathway is the main immune pathway during *M. gypseum* infection, which is consistent with the qRT-PCR results. 

## 3. Discussion

Currently, studies on *M. gypseum* infection are often used for antifungal drug screening locally and internationally, but a lack of research on the immune response after deep infection with *M. gypseum* has been observed [32]. Therefore, this study aims to fill the gaps in our knowledge of the immune response to *M. gypseum*.

### 3.1. Selection of the M. gypseum Infection Model

The establishment of a mouse model plays an important role in the study of fungal infections and the immune response generated by pathogenic fungi [29,33]. In general, the fungus infects mice via skin exposure, subcutaneous injection, intranasal injection, intraperitoneal injection, or intravenous injection. Innate and Th17 immune responses were studied in mice infected with *Cladosporium cladosporioides* via subcutaneous injection [34]. *Trichophyton rubrum*-infected mice were intraperitoneally inoculated to establish a murine model to study the effect of IL-17 on *T. rubrum* [28]. Ma et al. (2021) infected mice via tail vein injection and established a murine model to study the immune response mechanism after deep infection with *C. cladosporium* [35]. The immune response caused by fungal invasion has repeatedly been studied using a murine model of infection. Opportunistic pathogenic fungi infect animals when they are immunosuppressed. Current studies have established murine models using genetically altered mice [30], immunosuppressed mice [34], and vitamin D-deficient mice [36]. Therefore, in the present study, mice were infected via the tail vein, and a murine model was established to study the immune response mechanism in animals infected with *M. gypseum*.

### 3.2. Selection of Spore Infection Concentration of M. gypseum

Two concentrations, 8 × 10^6^ and 4 × 10^6^ CFU/mL, were used in the experiment. The results showed that both non-immunosuppressed mice at 8 × 10^6^ CFU/mL and at 4 × 10^6^ CFU/mL died on the first day after inoculation, and the survival rates in the first 10 days were only 40% and 6.67%, respectively. The survival rate of the non-immunosuppressed group at 4 × 10^6^ CFU/mL within 30 days was 100% and there was a significant decrease in body weight. Therefore, the non-immunosuppressed group with a dose of 4 × 10^6^ CFU/mL was selected as the optimal test parameter. Mariné et al. [37] indicated that mouse mortality increased as the number of *Trichosporon asahii* spores increased. Furthermore, Heather et al. [30] showed that immunosuppressed mice were more susceptible to infection and had higher mortality rates and weight loss. These results were consistent with those of our experiment.

### 3.3. Combined Analysis of Cytokine mRNA Expression and Flow Cytometry Results after M. gypseum Infection

The establishment of a protective immune response is associated with the establishment of an appropriate cytokine response [38]. To investigate the recruitment of immune cells at the site of infection, we evaluated the cytokine response *in situ* and combined the analysis with flow cytometry results. T cell subsets involved in the host immune response are crucial for an effective antifungal response. 

The Th1 pathway cytokine elicited by *M. gypseum* infection has a pro-inflammatory profile characterized by IFN-γ and IL-12. IFN-γ is secreted by Th1 cells, which enhances antigen presentation via macrophage activation, stimulates the innate immune system, mediates endothelial cells and lymphocytes interaction, and regulates cytokine profile expression and cell apoptosis [39]. Here, IFN-γ increased significantly at 2 dpi and then decreased rapidly to 14 dpi, but the instructive cytokine IL-12 was only significantly upregulated at 14 dpi, suggesting that the Th1-associated cytokine was weakly expressed during the immune response and that *M. gypseum* may induce delayed hypersensitivity (DTH) mediated by Th1 [40]. In addition, the flow cytometry results showed that Th1 cells exhibited a downward trend at 2 and 5 dpi. This further indicated that the Th1-associated cytokine was weakly expressed or gradually inhibited during the immune response after *M. gypsum* infection, and that a large amount of IFN-γ was produced by other innate immune cells.

The Th2 pathway cytokine produced due to the *M. gypseum* infection has a pro-inflammatory profile characterized by IL-13 and IL-4. Th2 cells, associated with specific airway hyperreactive diseases, produce a variety of other cytokines, such as IL-13, IL-4, IL-5, IL-9, and IL-25. IL-13 also leads to allergic asthma [26]. Zhang et al. reported that fungal exposure enhances allergen-driven Th2 responses, promoting severe allergic asthma. Fungal infections are related to environmental conditions and body immunity; therefore, mice infected with *M. gypsum* may be more sensitive to the environment. In our study, IL-13 and IL-4 were significantly upregulated in the lung tissue at 5 and 2 dpi, respectively, and then decreased. In addition, flow cytometry results showed that the number of Th2 cells was not statistically significant at 2 and 5 dpi. These results suggest that the Th2-associated cytokine was weakly expressed and may have been gradually inhibited during the immune response, and large amounts of IL-4 and IL-13 were produced by other innate immune cells.

The Th17 pathway cytokine elicited by the *M. gypseum* infection has a pro-inflammatory profile characterized by IL-17A, IL-17F, and IL-22. Cytokines including IL-6, IL-1β, IL-23, and TGF-β can activate Th17 cell differentiation and maturation. Differentiated and mature Th17 cells activate the corresponding immune and non-immune cells by secreting cytokines, such as IL-17, IL-21, IL-22, and granulocyte-macrophage colony stimulating factor (GM-CSF), to induce a variety of inflammatory and anti-pathogen reactions, thus eliminating the pathogenic fungi [11,41,42]. The Th17 pathway provides protective immunity against fungi, including *A. fumigatus* [43], *C. albicans* [44], and *T. rubrum* [28]. IL-17A, IL-17F, IL-21, and IL-22 can induce various inflammatory and antimicrobial responses in other cell types, including myeloid and epithelial cells [42,45]. IL-22 plays an important role in establishing skin and mucosa immunity [46]. Additionally, IL-17 regulates the expression of molecules with direct antimicrobial activity, such as β-defensins, and promotes the release of chemotactic factors, such as CCL20, consequently eliminating pathogenic fungi. Here, IL-17A, IL-17F, and IL-22 were significantly upregulated in the lung tissue at 2 and 5 dpi. Flow cytometry showed that the number of Th17 cells significantly increased. These results suggested that Th17 cells play an essential role in immunity against *M. gypseum* infection.

In addition, IL-22 can be produced by Th22 cells. IL-22 produced by Th22 cells is involved in protecting mucosal surfaces from tissue damage. It simultaneously synergizes with IL-17A and IL-17F to induce antimicrobial peptide expression in epithelial cells and mediate an early mucosal defense response to pneumonia-causing microorganisms in a mouse model [47,48]. Here, IL-22 expression was significantly upregulated in lung tissue at 2 and 5 dpi. Th22 cells may synergize with Th17 cells in promoting the immune effect of Th17 cells in *Microsporidium gypsum* infections.

The cytokine profile of Tregs produced due to *M. gypseum* infection is an anti-inflammatory profile characterized by TGF-β and IL-10. Treg cells produce the anti-inflammatory cytokines, IL-10 and TGF-β, during continuous antigen stimulation and induction of cytokines such as TGF-β, thereby, inhibiting the inflammatory response in the body [49]. Here, IL-10 was significantly upregulated in the lung tissue at 2 and 5 dpi. TGF-β was gradually upregulated from 2 dpi to 14 dpi. Significant upregulation of pro-inflammatory cytokines, including IFN-γ, IL-4, IL-6, IL-23, TNF-α, IL-1β, IL-17F, IL-17A, and IL-22, occurred at 2 and 5 dpi. These results suggest that days 2 and 5 marked the period of acute inflammation during the *M. gypseum* infection.

### 3.4. In-Depth Analysis of Th1, Th2, Th17, and Treg Signaling Pathways after M. gypseum Infection

Immunity in the body is a complex regulatory network. Measuring the expression of INF-γ, IL-4, and IL-22 as Th1, Th2, and Th17 in the experiment alone cannot fully represent the body’s immune response. Upon invasion by fungi, the innate immunity is rapidly activated, and the activation of antigen-presenting cells promotes the production of pro-inflammatory cytokines including TGF-β, IL-4, IL-23, IL-6, IL-12, and IFN-γ. DCs produce IL-12, IL-4, and IL-10 to induce Th1 and Th2 immune responses [50]. In the early infection stages, the body resists fungal infection via a wide range of innate immune effector mechanisms and a large number of cytokines are produced by innate immune cells.

In addition, after a fungus invades the body, receptors are stimulated to recognize and activate various downstream signaling pathways to produce various cytokines, thereby initiating specific immune regulation. In the Th1 pathway, IL-12 and IFN-γ are the primary expression factors co-regulated by T-bet and IL-12Rb. In the Th2 pathway, IL-4 and IL-13 are the primary expression factors. The Th2 cytokine c-Maf cooperates with NFATc1 to increase the expression of IL-4 [51]. Tfh cells can differentiate into IL-4/IL-13 twin Th2 cells that recruit eosinophils to the lungs [52,53]. In the Th17 pathway, STAT3 is phosphorylated by the JAK-STAT signaling pathway driven by IL-23, IL-6, and IL-21; low doses of TGF-β and IL-1β can regulate the expression of RORγ and RORα, and then regulate Th17, the main expression factors IL-17A, IL-17F, IL-21, and IL-22 [54]. Therefore, IFN-γ, IL-4, and IL-22 expression is the result of the joint regulation of various cytokines and signaling pathways.

Except for cytokine and signaling pathway regulation, Th1, Th2, Th17, and Treg cell differentiation restrict one another and affect the expression of IFN-γ, IL-4, and IL-22. In the Th1 pathway, upregulation of T-bet inhibits the expression of GATA3, promotes Th1 differentiation, and antagonizes Th2 [55]. In the Th2 pathway, IL-4 inhibits the differentiation of Th1. Both Th1 and Th2 cells can secrete cytokines to promote their own differentiation and inhibit the other’s differentiation. TGF-β induces the differentiation of Treg cells, inhibits the differentiation of Th1 and Th2 cells, and promotes the differentiation of Th17 cells [56,57]. The growth and development of Th17 cells require the support of IL-23 and TGF-β and are negatively regulated by IFN-γ and IL-4, but they can resist the inhibitory effect of IFN-γ and IL-4 when TH17 cells grow and mature.

Therefore, we speculate that the results of flow cytometry showed that there were no significant changes in Th1 and Th2 cells. This is because Treg and Th17 cells negatively regulated Th1 and Th2 cells at 2 and 5 dpi, so that the expression of Th1 and Th2 pathways was weak, and a large number of cytokines, such as IL-12, IFN-γ, IL-4, IL-9, and IL-13, were produced by other innate immune cells, causing the mRNA expression of fluorescence quantitative results to be significant. These results reflect the specific pathogenesis of *M. gypseum* infections in vivo.

In the present study, qRT-PCR was used to detect changes in expression at the mRNA level. It is a complex process from mRNA transcription to protein translation. Firstly, the time and site of transcription and translation of eukaryotic gene expression are separated in time and space. Second, further multi-level processing and modification are required after transcription and translation. Therefore, the transcriptional and translational levels are not necessarily exactly the same. Next, the body’s immune response to *M. gypseum* should be further analyzed at the transcriptomic, proteomic, and single-cell transcriptome levels.

### 3.5. Immune Response Patterns of M. gypseum Infection In Vivo

Antigen-presenting cells (APCs) are immune cells that process and present antigens to T cells, including macrophages and DCs. The inflammatory response observed in the lungs of mice infected with *M. gypseum* is characterized by an inflow of immune cells, such as PMNs, macrophages, and DCs. Neutrophil extracellular traps (NETs) are essential components of neutrophils in antifungal immunity. NETs mediate host defense by trapping and killing microorganisms when the body is infected [58]. DCs regulate the distribution of neutrophils in the blood and peripheral organs [59]. The deep lung tissue contains a well-developed innate immune system, including alveolar macrophages. Alveolar macrophages are usually at rest and self-regulate to form an appropriate immune response to protect lung tissue [60]. Here, large numbers of PMNs (neutrophils +), DCs (CD11c+), and macrophages (CD54+) were recruited to the alveoli septa and bronchi of mice infected with *M. gypseum*. Dendritic cells, macrophages, and neutrophils play essential roles in the initiation of antifungal immune responses. 

DCs and macrophages express various receptors on their surfaces, which play important roles in initiating antifungal immune responses. PRRs closely related to antifungal immune responses mainly include TLR-2, TLR-4, Dectin-1, and Dectin-2 [22]. The transcriptional expression levels of pulmonary receptors TLR-2, TLR-4, and Dectin-1 were significantly increased after intravenous injection of *M. gypseum* in mice, suggesting that TLR-2, TLR-4, and Dectin-1 are involved in the recognition, internalization, and subsequent activation of immune responses against fungi. 

**Figure 10:**
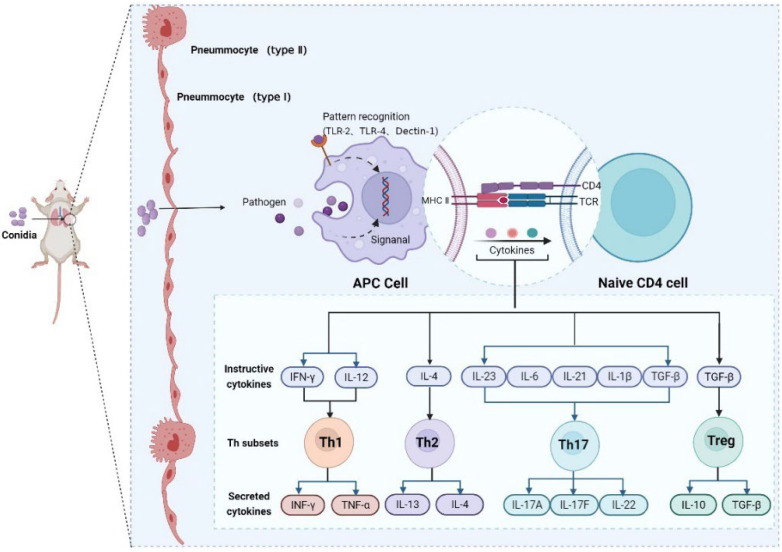
Patterns of immune responses in mice infected with *M. gypseum*. APCs recognize *M. gypseum* conidia through receptors, triggering innate immune response and secreting pro-inflammatory cytokines. The pro-inflammatory cytokines induce T cells to proliferate and differentiate into Th1, Th2, Th17, and Treg cells, and produce corresponding cytokines to regulate immune responses. TCR activate T cells after binding to antigen-carrying MHC II, and finally trigger a series of immune responses, such as antibody production.

This study investigated the pattern of immune responses in mice infected with *M. gypseum in vivo* (Figure 10). After the *M. gypseum* conidia suspension entered the mice via the tail vein, it mainly colonized and damaged the lung tissue, causing an immune response in the lung tissue. The APC cell was recognized, ingested, and processed by *M. gypseum* conidia via endocytosis or the RPPs (TLR-2, TLR-4, and Dectin-1) receptor to generate corresponding signals. The signal promoted the release of pro-inflammatory cytokines, such as IL-12, IL-4, and IL-6, through Dectin-1 and TLRs receptors and induced the T cell receptor (TCR) to bind to MHC II with antigens, thereby activating naïve CD4 cells that polarized into Th1, Th2, Th17, and Treg cells and produced corresponding cytokines to regulate the immune response. In our study, the adaptive immune response generated by mice infected with *M. gypseum* occurred mainly through the Th17 pathway, and Tregs played an important role in inhibiting inflammation. 

## 4. Materials and Methods

### 4.1. Fungal Strain and Conidia Preparation

*M. gypseum* (preserved at Sichuan Agriculture University) was originally isolated from a giant panda suffering from dermatomycosis, stored at −80 °C with 15% (*v*/*v*) glycerol, and subcultured twice on Sabouraud dextrose agar (SDA) at 25 °C before use. The strain was grown on SDA at 25 °C for 9 days, scraped with sterile phosphate buffered saline (PBS) from the SDA medium, transferred to a sterile centrifuge tube, and allowed to stand for 30 min to aspirate the intermediate liquid. Conidia were washed with PBS and diluted to 4 × 10^6^ CFU/mL and 8 × 10^6^ CFU/mL using a hemocytometer for experimental infection.

### 4.2. Animals

Specific pathogen-free, 6-week-old female mice of the KM strain (Dashuo Experimental Animals Co., Ltd. Chengdu, China) were housed in a sterile environment with free access to food and water during the entire study period. The mice were euthanized by cervical dislocation, decapitation, and dissected to observe lesions when required. All animal experiments were approved by the Institutional Animal Care and Use Committee of Sichuan Agricultural University (permit number: DYY-S20171209).

### 4.3. Determination of Weight and Mortality

Some mice were immunosuppressed with a single intraperitoneal injection of 200 mg/kg cyclophosphamide (Jiangsu Hengrui Pharmaceutical Co., Ltd. Lianyungang, China) a day before intravenous infection with *M. gypseum* [37]. To conduct weight and mortality (n = 15 mice per group), flow cytometry (6 mice per group), and other experiments (n = 8 mice per group), mice were randomly selected from each group. Experimental infections were as follows: non-immunosuppressed mice in the control group were intravenously injected with 0.25 mL of PBS; non-immunosuppressed mice in the experimental group were intravenously injected with 0.25 mL (4 × 10^6^ CFU/mL); non-immunosuppressed mice in the experimental group were intravenously injected with 0.25 mL (8 × 10^6^ CFU/mL); immunosuppressed control mice were intravenously injected with 0.25 mL); and immunosuppressed mice in the experimental group were intravenously injected with 0.25 mL (4 × 10^6^ CFU/mL). The follow-up observations of status, weight of the empty stomach, and mortality were recorded daily after infection. 

### 4.4. Determination of Organ Index 

The organ index was determined by injecting 0.25 mL (4 × 10^6^ CFU/mL) *M. gypseum* into the non-immunosuppressed mice, and the body and tissue weight of the animals were measured at 2, 5, and 9 dpi. The mice were weighed on an empty stomach. The heart, liver, spleen, lungs, and kidneys were weighed immediately after removal. The OI was calculated using the following formula: OI = (tissue weight/body weight) × 100.

### 4.5. Tissue Fungal Burden Analysis

Tissue fungal burden was determined by injecting 0.25 mL (4 × 10^6^ CFU/mL) *M. gypseum* into non-immunosuppressed mice, and the heart, liver, spleen, lungs, and left kidney of the animals were weighed at 2, 5, and 9 dpi, respectively. The tissues were immediately weighed after removal, transferred to 4 mL of PBS, mechanically homogenized using a mortar, and serially diluted to 10^−1^, 10^−2^, and 10^−3^. Aliquots of undiluted and diluted homogenates (100 µL) were plated onto SDA plates and incubated at 25 °C for 72 h. Recovered colonies were counted, multiplied by the dilution factor, and expressed as a mean log.

### 4.6. Histology

The histology was determined by injecting 0.25 mL (4 × 10^6^ CFU/mL) into the non-immunosuppressed mice, and the liver, spleen, lungs, and left kidney tissues collected at 2, 5, 9, and 14 dpi were fixed in 10%(*v*/*v*) neutralized buffered formalin and paraffin-embedded for routine processing. The samples were stained with HE for histopathological evaluation or periodic acid-Schiff (PAS) staining to assess fungal invasion in the tissue structures.

### 4.7. Quantitative Real Time-Polymerase Chain Reaction (qRT-PCR)

RNA extraction was determined by injecting 0.25 mL (4 × 10^6^ CFU/mL) *M. gypseum* into the non-immunosuppressed mice, and lung tissue was collected at 2, 5, 9, and 14 dpi. Total RNA was isolated using TRIzol reagent, according to the manufacturer’s instructions (Invitrogen, Beijing, China). The cDNA template was synthesized from RNA via reverse transcription using a PrimeScript™ RT Reagent Kit with gDNA Eraser (TaKaRa, Dalian, China).

The primers for TLR-2, TLR-4, and Dectin-1 were designed using DNAStar (Version 6.0) and Primer (Version 5.6) software according to the sequence in GenBank (Table 1). The sequences of oligonucleotide primers have already been published: 12s rRNA, β-actin, interleukin (IL)-6, IL-1β, TNF-α, and IL-22; IFN-γ, IL-4, and IL-17A; IL-9 [61]; IL-13; TGF-β [62]; IL-23; and IL-17F [63] (Table 1). Each qPCR contained 10 mL TB Green® Premix Ex Taq™ II (Tli RNaseH Plus) (Takara, Dalian, China), 0.1 mg cDNA, and 0.4 μM of each gene-specific primer (Table 1) in a final volume of 20 mL and was performed on a CFX96 real-time PCR system (BioRad, Hercules, CA, USA). β-Actin and 12s rRNA were used as the internal reference genes. The cycle threshold (Ct) of the internal reference was calculated according to the following formula: Ct =$\sqrt[{2}]{Ct\left(12s\;rRNA)\times Ct(\beta -actin\right)}$. qRT-PCR data were analyzed using the 2^–ΔΔCt^ method to calculate the relative expression levels of cytokine and receptor mRNA in lung tissue at 2, 5, 9, and 14 dpi compared to day 0. All assays were performed in triplicate. 

### 4.8. Immunofluorescence Staining

Immunofluorescence was determined by injecting 0.25 mL (4 × 10^6^ CFU/mL) *M. gypseum* into the non-immunosuppressed mice, and the lung tissues collected at 2, 5, and 9 dpi were immersed in 10% paraformaldehyde embedding medium (Wuhan Junjie Electronics Co., Ltd. Wuhan, China). After two incubations in xylene, the sections were dehydrated in gradient ethanol with 85% and 75% ethanol, respectively. Samples were then washed in distilled water. The slides were immersed in ethylenediaminetetraacetic acid (EDTA) antigen retrieval buffer (pH 8.0) and maintained at a sub-boiling temperature (98 °C) for 8 min, allowed to stand for 8 min (95 °C~98 °C), and followed by another sub-boiling temperature for 7 min. Slides were washed three times with PBS (pH 7.4), the notable liquid was eliminated, and the objective tissue was marked with a liquid blocker pen. A spontaneous fluorescence quenching reagent was added and incubated for 5 min (15 °C~25 °C). The sample was then washed under running tap water, and the marked tissue was covered with 3% bovine serum albumin (BSA) to block non-specific binding for 30 min. 

Slides were incubated with the primary antibody (diluted with PBS) overnight at 4 °C. The slides were washed three times with PBS (pH 7.4), the objective tissue was covered with secondary antibody (appropriately responding to the primary antibody in species), and incubated at room temperature (15 °C~25 °C) for 50 min in the dark. The cells were then incubated with a DAPI solution at room temperature (15 °C~25 °C) for 10 min. The excess liquid was discarded and a coverslip was mounted with an anti-fade mounting medium. Images were obtained using a microscope and were collected using a fluorescence microscope (Leica, DM2000, DFC450C (camera)). The characteristics of the primary and secondary antibodies used are listed in Table 2.

### 4.9. Flow Cytometry

After the lung tissue was minced by mechanical methods, type I collagenase and DNase were added and the tissue was allowed to digest at 37 °C for 40 min, whereafter it was filtered through a 300-mesh filter cloth, centrifuged at 300× *g* for 5 min, the supernatant was discarded, and the cell concentration was adjusted to 10^6^ cells/mL with PBS solution. Cell suspensions (100 μL) were placed in a flow tube, 1 µg of CD3 and CD4 surface-labeled antibodies was added, they were incubated at 4 °C for 30 min, then 2 mL of PBS solution was added for washing once, and the supernatant was discarded after completion. Foxp3 Fixing/Breaking Membrane Working Solution (eBioscience, San Diego, CA) (1 mL) was added to each tube, mixed by pulse vortexing, and incubated at room temperature (15 °C~25 °C) for 50 min. A 1X membrane breaking solution (2 mL) was added to each tube and the sample was centrifuged at 400—600× *g* for 5 min at room temperature. The supernatant was discarded, and this step was repeated once. Fluorescence-conjugated antibody (1 μg) was added to detect intracellular antigens (IL4, IFN-γ, and IL-17A) (BD Biosciences) and incubated at room temperature for more than 30 min. A 1X membrane-breaking solution (2 mL) was added to each tube and centrifuged at 400—600× *g* for 5 min at room temperature. The supernatant was discarded, and this step was repeated once. The PBS (400 µL) was added to resuspend the cells. Detection was performed using a flow cytometer (ZE5, Bio-Rad, Hercules, CA, USA) and the data were analyzed.

### 4.10. Statistical Analysis

The weights of mice and tissues, OI values, expression levels of receptors and cytokine genes, and flow cytometry at different time points compared with day 0 were analyzed using Duncan’s test. Tissue fungal burden counts of the evaluated organs at different time points were statistically analyzed using Dunn’s multiple comparison test. All statistical analyses were performed using GraphPad Prism 5.01 (GraphPad Software Inc., La Jolla, CA, USA), with *p* < 0.05 indicating a statistically significant differences (*p* < 0.01 indicating a highly significant difference, and *p* < 0.001 indicating an extremely high significant difference).

## 5. Conclusions

*M. gypseum* induces an inflammatory reaction in the early stages of infection, and the lung is the main target of colonization and site of injury. The receptors Dectin-1, TLR-2, and TLR-4 play a role in the recognition of *M. gypseum*, and adaptive immunity mainly occurs via the Th17 pathway.

## Figures and Tables

**Table 1:** Sequences of oligonucleotide primers used for quantitative RT-PCR analysis.

Target Gene	Primer Sequence (5′ > 3′)		PCR Product Size (bp)
	Forward	Reverse	
12s rRNA	GGAAGGCATAGCTGCTGGAGGT	CGATGACATCCTTGGCCTGA	164
β-Actin	TTCCAGCGTTCCTTCTTGGGT	GTTGGCATAGAGGTGTTTACG	90
IL-6	GTTCTCTGGGAAATCGTGGA	TGTACTCCAGGTAGCTATGG	208
IL-1β	TTGACGGACCCCAAAAGATG	AGAAGGTGCTCATGTCCTCA	204
TNF-α	TCTCATCAGTTCTATGGCCC	GGGAGTAGACAAGGTACAAC	164
IL-22	GGCCAGCCTTGCAGATAACA	GCTGATGTGACAGGAGCTGA	220
IL-10	AGCCGGGAAGACAATAACTG	CATTTCCGATAAGGCTTGG	189
TGF-β	TGCCCTCTACAACCAACACA	GTTGGACAACTGCTCCACCT	277
IL-23	TGTGCCCCGTATCCAGTGT	CGGATCCTTTGCAAGCAGAA	81
IL-17F	CTGAGGCCCAGTGCAGACA	GCTGAATGGCGACGGAGTT	189
IL-17A	GCTCCAGAAGGCCCTCAGA	AGCTTTCCCTCCGCATTGA	187
IFN-γ	AAAGACAATCAGGCCATCAG	TGGGTTGTTGACCTCAAACT	129
IL-4	CATCGGCATTTTGAACGAG	TTGGAAGCCCTACAGACGAG	120
IL-9	CTGATGATTGTACCACACCGTGC	GCCTTTGCATCTCTGTCTTCTGG	237
IL-13	AGACTCCCCTGTGCAACGGCA	GGAGACCGTAGTGGGGGCCTT	167
TLR-2	CGACATCCATCACCTGACTCTTC	GCCTCGGAATGCCAGCTTCTTC	182
TLR-4	ACAAGGCATGGCATGGCTTACAC	TGTCTCCACAGCCACCAGATTCTC	125
Dectin-1	CCCTCCAAGGCATCCCAAAC	CCTAGCTGGGAGCAGTGTCT	140

**Table 2:** Antibodies used for immunofluorescence staining.

Primary Antibody	Target	Secondary Antibody	Source
Rat antimouse CD54 (ICAM-I) biotin	Macrophages	Goat Anti-rat CY3	eBioscience (San Diego, CA, U.S.A.)
Hamster antimouse CD11c biotin	DCs	Goat Anti-rat CY3	eBioscience
Rat antimouse neutrophil	PMNs	Goat Anti-rat CY3	Abcam (Cambridge, U.K.)

DCs, dendritic cells; ICAM, intercellular adhesion molecule; PMNs, polymorphonuclear neutrophils.

## Data Availability

The datasets supporting the conclusions of this study are included in the article.

## Supplementary Materials

The following supporting information can be downloaded at: https://www.mdpi.com/article/10.3390/ijms231912037/s1.
