# Supplementary material for: Microsporum gypseum Isolated from Ailuropoda melanoleuca Provokes Inflammation and Triggers Th17 Adaptive Immunity Response
Source: Int J Mol Sci. 2022 Oct 10;23(19):12037. doi: 10.3390/ijms231912037 (PMC9570494; doi:10.3390/ijms231912037)
Supplement: Supplementary file 1 [file ijms-23-12037-s001.zip › ijms-1859616-supplementary.pdf]

International Journal of
Molecular Sciences

Article

Microsporum gypseum Isolated from *Ailuropoda melanoleuca* Provokes Inflammation and Triggers Th17 Adaptive Immunity Response

Xiaoping Ma [1,†], Zhen Liu [1,†], Yan Yu [1], Yaozhang Jiang [1,‡], Chengdong Wang [2], Zhicai Zuo [1], Shanshan Ling [2], Ming He [1,2], Sanjie Cao [1], Yiping Wen [1], Qin Zhao [1], Rui Wu [1], Xiaobo Huang [1], Zhijun Zhong [1], Guangneng Peng [1] and Yu Gu [3,*]

[1] Key Laboratory of Animal Disease and Human Health of Sichuan Province, College of Veterinary Medicine, Sichuan Agricultural University, Chengdu 611130, China
[2] China Conservation and Research Center for the Giant Panda, Chengdu 611800, China
[3] College of Life Sciences, Sichuan Agricultural University, Chengdu 611130, China
* Correspondence: guyu632@sicau.edu.cn; Tel.: +086-18190681226
† These authors contributed equally to this work.
‡ Current address: Bioengineering Department, Sichuan Water Conservancy Vocational College, Chengdu 611231, China.

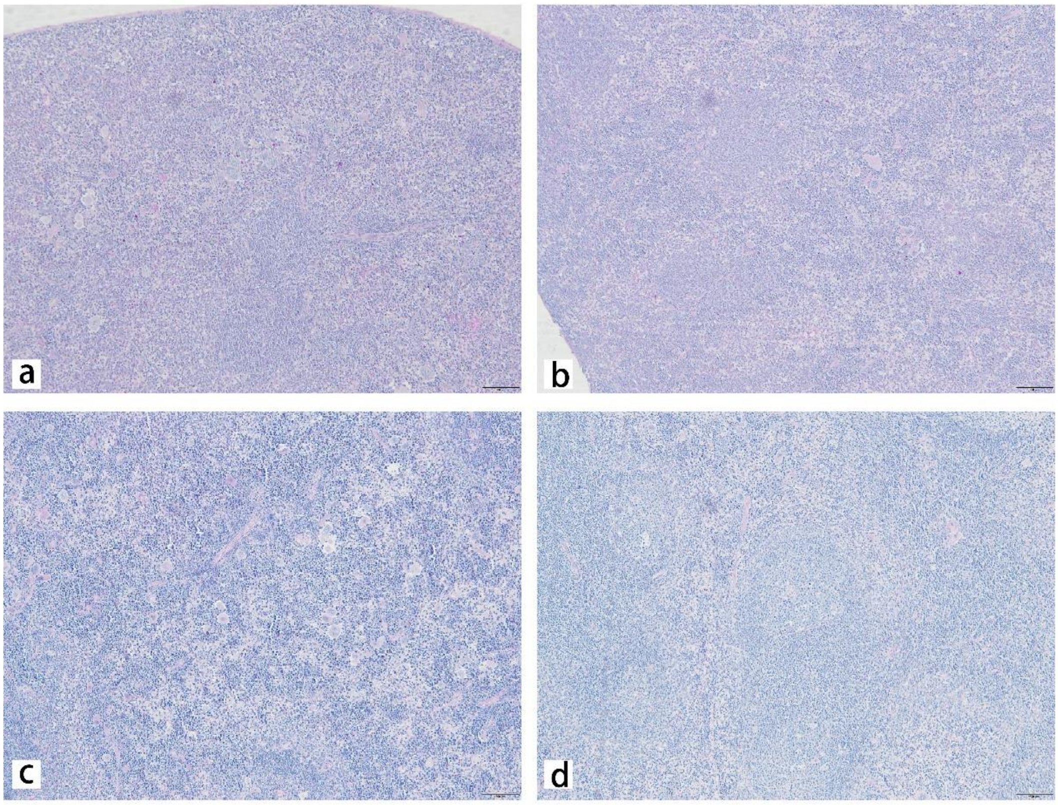

Supplementary Figure S1 A Representative PAS-stained sections of the spleen from mice on days 2, 5, 9, and 14 after infection; i.v. infected with *M. gypseum* (4×10^6 CFU/mL). Scale bars, 500 µm.

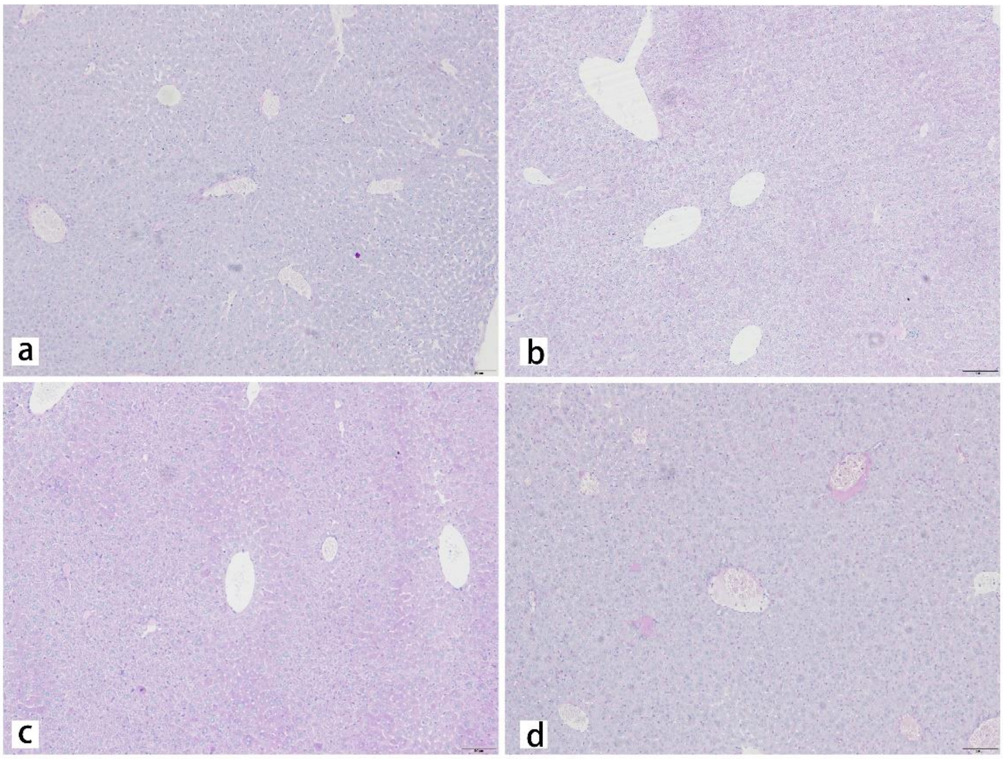

Supplementary Figure S1B Representative PAS-stained sections of the liver from mice on days 2, 5, 9, and 14 after infection; i.v. infected with M. gypseum (4×106 CFU/mL). Scale bars, 500 μm.

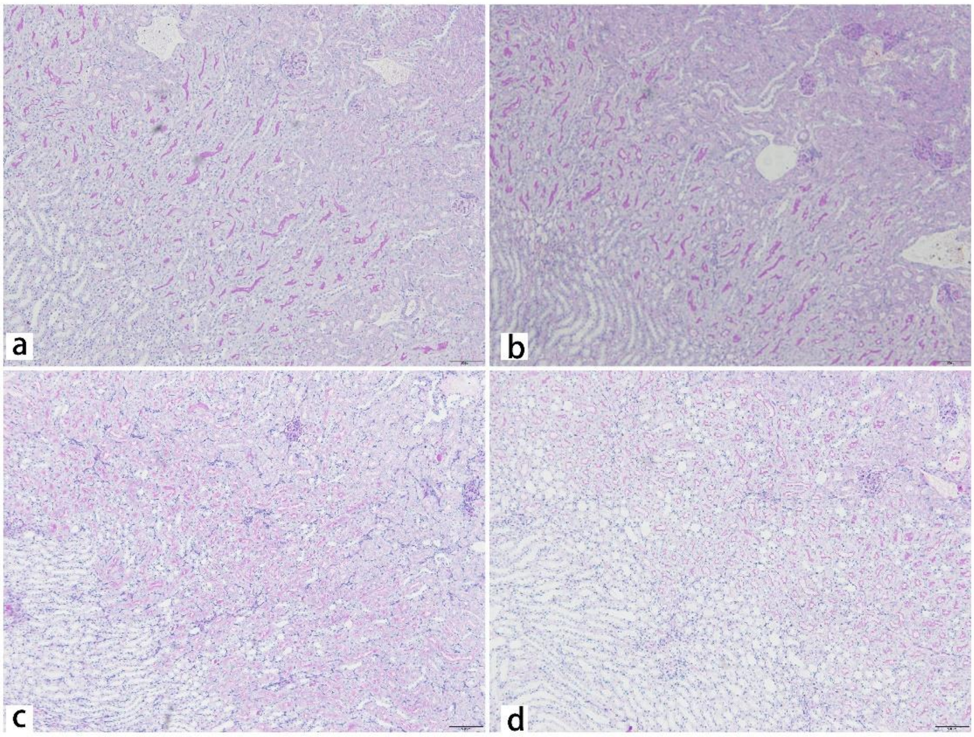

Supplementary Figure S1 C Representative PAS-stained sections of the spleen(A), liver(B), kidney(C), and heart(D) from mice on days 2, 5, 9, and 14 after infection; i.v. infected with *M. gypseum* ($4×10^6$ CFU/mL). Scale bars, 500 μm.

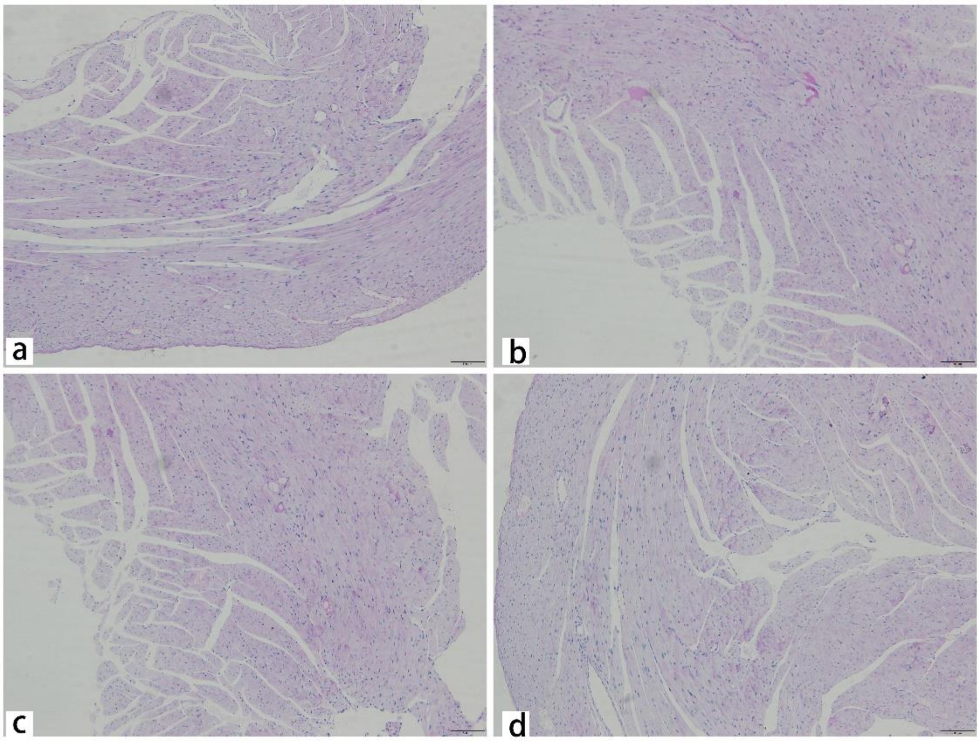

Supplementary Figure S1D. Representative PAS-stained sections of the spleen(A), liver(B), kidney(C), and heart(D) from mice on days 2, 5, 9, and 14 after infection; i.v. infected with *M. gypseum* (4×10^6 CFU/mL). Scale bars, 500 μm.

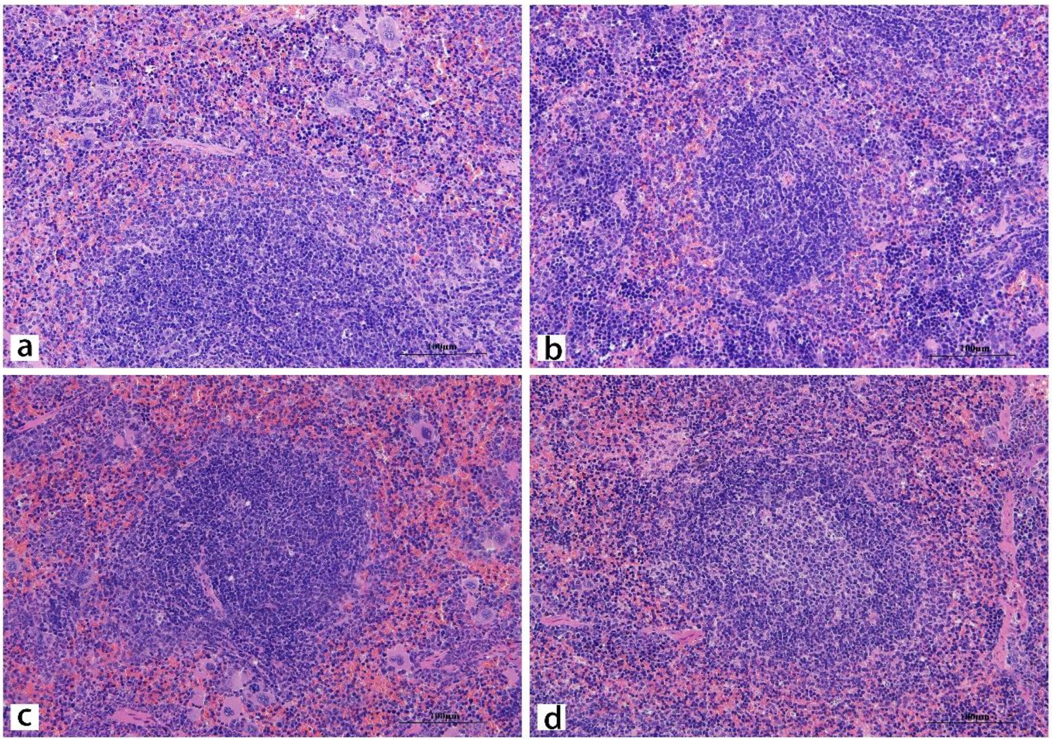

Supplementary Figure S2 A Representative HE-stained sections of the spleen from mice on days 2, 5, 9, and 14 after infection; i.v. infected with *M. gypseum* (4×10^6 CFU/mL). Scale bars, 100 μm.

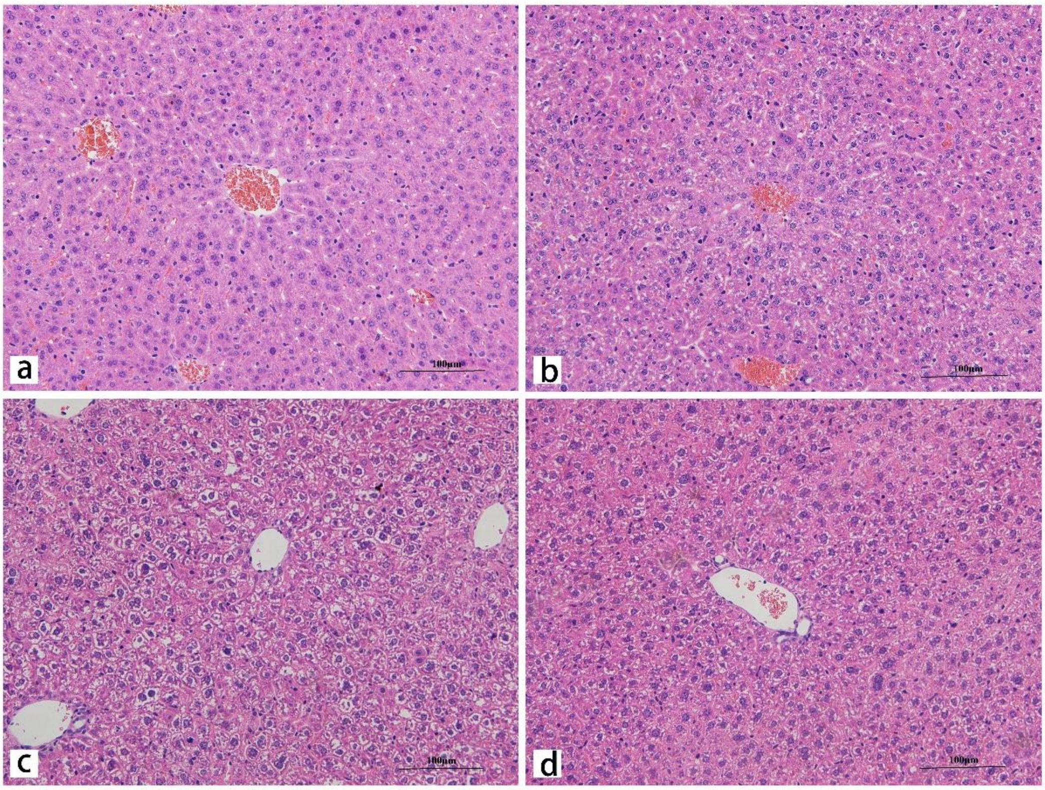

Supplementary Figure S2B Representative HE-stained sections of the liver from mice on days 2, 5, 9, and 14 after infection; i.v. infected with *M. gypseum* (4×10^6 CFU/mL). Scale bars, 100 μm.

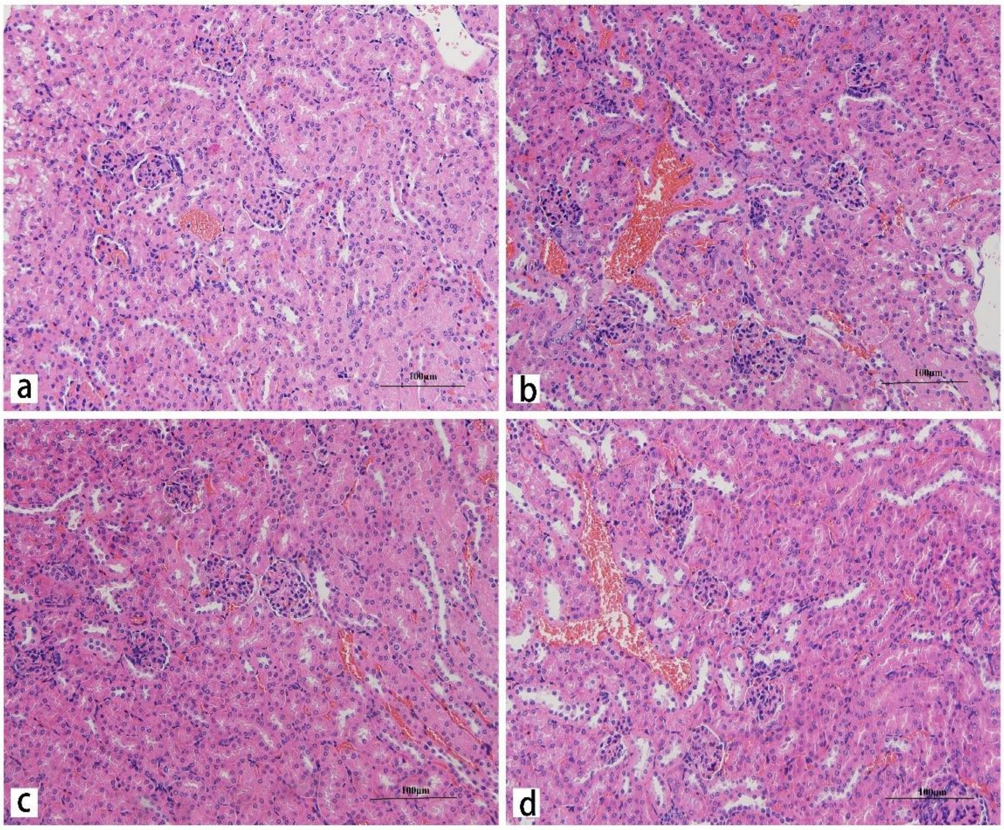

Supplementary Figure S2 C Representative HE-stained sections of the kidney from mice on days 2, 5, 9, and 14 after infection; i.v. infected with *M. gypseum* (4×10^6 CFU/mL). Scale bars, 100 μm.
